# More rapid blood interferon α2 decline in fatal versus surviving COVID-19 patients

**DOI:** 10.3389/fimmu.2023.1250214

**Published:** 2023-11-21

**Authors:** Candie Joly, Delphine Desjardins, Raphael Porcher, Hélène Péré, Thomas Bruneau, Qian Zhang, Paul Bastard, Aurélie Cobat, Léa Resmini, Olivia Lenoir, Laurent Savale, Camille Lécuroux, Céline Verstuyft, Anne-Marie Roque-Afonso, David Veyer, Gabriel Baron, Matthieu Resche-Rigon, Philippe Ravaud, Jean-Laurent Casanova, Roger Le Grand, Olivier Hermine, Pierre-Louis Tharaux, Xavier Mariette

**Affiliations:** ^1^ Université Paris-Saclay, INSERM, CEA, Center for Immunology of Viral, Auto-immune, Hematological and Bacterial diseases (IMVA-HB/IDMIT), UMR1184, Le Kremlin Bicêtre, France; ^2^ Université de Paris, Center of Research in Epidemiology and Statistics (CRESS), INSERM, INRAE, AP-HP, Hôpital Hôtel-Dieu, Paris, France; ^3^ Sorbonne Université and Université de Paris, INSERM, Functional Genomics of Solid Tumors (FunGeST), Centre de Recherche des Cordeliers, Paris, France; ^4^ Service de Microbiologie (Unité de virologie), Assistance Publique Hôpitaux de Paris-Centre (AP-HP-Centre), Hôpital Européen Georges Pompidou, Paris, France; ^5^ Laboratory of Human Genetics of Infectious Diseases, Necker Branch, INSERM U1163, Necker Hospital for Sick Children, Paris, France; ^6^ University of Paris, Imagine Institute, Paris, France; ^7^ St. Giles Laboratory of Human Genetics of Infectious Diseases, Rockefeller Branch, The Rockefeller University, New York, NY, United States; ^8^ Université de Paris, INSERM, Paris Cardiovascular Center (PARCC), Paris, France; ^9^ Université Paris-Saclay, Faculté de Médecine, Le Kremlin-Bicêtre, France; ^10^ AP-HP, Centre de Référence de l’Hypertension Pulmonaire, Service de Pneumologie et Soins Intensifs Respiratoires, Hôpital Bicêtre, Le Kremlin-Bicêtre, INSERM UMR999, Hôpital Marie Lannelongue, Le Plessis Robinson, France; ^11^ Université Paris-Saclay, Assistance Publique-Hôpitaux de Paris, Hôpital Bicêtre, Centre de Ressource Biologique Paris-Saclay, Le Kremlin Bicêtre, France; ^12^ Université Paris-Saclay, Assistance Publique-Hôpitaux de Paris, Hôpital Paul Brousse, Laboratoire de Virologie, Villejuif, France; ^13^ Centre of Research in Epidemiology and Statistics (CRESS), Université de Paris, INSERM, Hôpital Saint Louis, Paris, France; ^14^ Howard Hughes Medical Institute, New York, NY, United States; ^15^ Université de Paris, Institut Imagine, INSERM UMR1183, Paris, France; ^16^ Assistance Publique-Hôpitaux de Paris, Hôpital Necker, Département d’Hématologie, Paris, France; ^17^ Assistance Publique-Hôpitaux de Paris, Hôpital Bicêtre, Service de Rhumatologie, Le Kremlin Bicêtre, France

**Keywords:** COVID-19, pneumonia, SARS-CoV-2, type I interferon, prospective study

## Abstract

**Background:**

The clinical outcome of COVID-19 pneumonia is highly variable. Few biological predictive factors have been identified. Genetic and immunological studies suggest that type 1 interferons (IFN) are essential to control SARS-CoV-2 infection.

**Objective:**

To study the link between change in blood IFN-α2 level and plasma SARS-Cov2 viral load over time and subsequent death in patients with severe and critical COVID-19.

**Methods:**

One hundred and forty patients from the CORIMUNO-19 cohort hospitalized with severe or critical COVID-19 pneumonia, all requiring oxygen or ventilation, were prospectively studied. Blood IFN-α2 was evaluated using the Single Molecule Array technology. Anti-IFN-α2 auto-Abs were determined with a reporter luciferase activity. Plasma SARS-Cov2 viral load was measured using droplet digital PCR targeting the Nucleocapsid gene of the SARS-CoV-2 positive-strand RNA genome.

**Results:**

Although the percentage of plasmacytoid dendritic cells was low, the blood IFN-α2 level was higher in patients than in healthy controls and was correlated to SARS-CoV-2 plasma viral load at entry. Neutralizing anti-IFN-α2 auto-antibodies were detected in 5% of patients, associated with a lower baseline level of blood IFN-α2. A longitudinal analysis found that a more rapid decline of blood IFN-α2 was observed in fatal versus surviving patients: mortality HR=3.15 (95% CI 1.14–8.66) in rapid versus slow decliners. Likewise, a high level of plasma SARS-CoV-2 RNA was associated with death risk in patients with severe COVID-19.

**Conclusion:**

These findings could suggest an interest in evaluating type 1 IFN treatment in patients with severe COVID-19 and type 1 IFN decline, eventually combined with anti-inflammatory drugs.

**Clinical trial registration:**

https://clinicaltrials.gov, identifiers NCT04324073, NCT04331808, NCT04341584.

## Introduction

COVID-19 is a respiratory disease induced by a novel coronavirus (SARS-CoV-2), causing substantial morbidity and mortality (almost 18 million deaths in January 2023). Most people with COVID-19 have no or only mild clinical manifestations, including upper respiratory disease and moderate (non-hypoxemic) pneumonia. Still, approximately 10% to 15% may require hospitalization and oxygen support, and 3% to 5% eventually are admitted to an intensive care unit (ICU) (1–3). Age and comorbidities such as cardiovascular and respiratory diseases, hypertension, and diabetes contributes to disease severity in COVID-19 (4, 5). In addition to these pre-existing factors, several studies demonstrate that excessive inflammation following SARS-CoV-2 infection is associated with higher risk of developing severe COVID-19 (6). This is consistent with the elevated pro-inflammatory cytokines/chemokines (TNF, IL-6, IL-1b, IL-8) serum levels (7, 8) elevated level of C-reactive protein (CRP), D-dimer and ferritin, and lymphopenia observed in patients with severe diseases (9).

Among other immune dysfunction, type I interferons (IFN) response have emerged as a critical determinant of COVID-19 severity. Type I IFN are a family of anti-viral and immunomodulatory cytokines, which includes 13 IFN-α subtypes, IFN-β, and other poorly defined subtypes (IFN-ϵ, IFN-ω, IFN-κ, IFN-τ, IFN-δ and IFN-ζ), secreted by the host during most viral infections (10) and mainly produced by a subset of dendritic cells called plasmacytoid dendritic cells (pDC). Unlike other viral infections, SARS-CoV-2 induces little amounts of IFN, primarily type I (α and β) and type III (Λ) (11, 12). However, the question persists in determining if the level or the kinetic of type I and III IFN response predicts a worst outcome (13). Most severe and critical patients are characterized by low amounts of circulating type I IFN and a diminished IFN blood signature (14, 15). Furthermore, inborn errors of TLR3- and TLR7-dependent type I IFN immunity genes (16, 17) and a high prevalence of anti-type I IFN neutralizing auto-antibodies (auto-Abs) (18, 19) have documented that impaired type I IFN response is involved in critical cases. This latter finding was markedly confirmed in a large cohort where auto-Abs neutralizing low concentrations of IFN-α and/or -ω (100 pg/mL, in 1/10 dilutions of plasma) were found in 13.6% of 3,595 patients with critical COVID-19 (19). Lastly, some studies have found a decreased proportion of pDC in the blood of patients with severe or critical COVID-19, which could fit with the poor IFN response (14, 15).

While a poor IFN response at the onset of infection is widely accepted as a driver of severe COVID-19, some authors suggest that a delayed and prolonged increased interferon response may be detrimental and pathogenic (20). Type I IFN could be beneficial at the early stage of SARS-CoV-2 infection (21) but could also have a deleterious effect at later stages of the disease by favoring hyperactivation of lung macrophages (22). Understanding and solving this discrepancy in the literature is key to the optimal design of therapeutic strategies, including the timing and route of recombinant IFNs administration. In addition, the relationship between IFN and plasma viral load, another marker of disease severity (23, 24) has to be evaluated.

To characterize the dynamics of the IFN response, we performed a prospective and longitudinal study assessing in patients with severe and critical COVID-19 the amount of circulating IFN-α2 using the Simoa technology and SARS-CoV-2 viral load using digital PCR. We found that a more rapid decline of IFN-α2 in the first 6 days upon admission was observed in fatal versus surviving patients. SARS-CoV-2 RNAaemia was also significantly associated with the risk of death.

## Materials and methods

### Design of the study

One hundred and forty patients from the CORIMUNO-19 cohort (25) were included and divided into two groups: group 1: patients with severe pneumonia requiring at least 3L/min of oxygen (n=81), and group 2: patients with critical pneumonia requiring non-invasive or mechanical ventilation in intensive care unit (ICU) (n=59). These patients were included in three randomized clinical trials, one comparing usual care (UC) to UC plus an anti-IL-6 receptor antagonist (sarilumab), the second one comparing usual care (UC) to UC plus another anti-IL-6 receptor antagonist (tocilizumab), the last one comparing UC to UC plus an IL-1 antagonist (anakinra). The blood level of IFN-α2 was monitored longitudinally in all patients, and plasmatic viral load in almost all. PBMC were collected at baseline in one site (Bicêtre hospital) in 14 patients included in the Anakinra trial (26).

In order to determine whether IFN-α2 variation during disease evolution may have a predictive value, we collected samples at entry in the hospital (D1), day 3 (D3), D6, and D14 in most of the patients to assess blood IFN-α2. At D6, 105 of the patients had a second measurement. Among the 27 patients without any measurement à D6, 4 were dead (three in group 1 and two in group 2), and 23 missed the second sample (all in group 1).

During the 90-day follow-up of the study, 29 patients died, including 6 between D1 and D6. Since none of these drugs directly interfered with type I IFN production, we chose in the main analysis to pool the overall population regardless of the treatment.

### The CORIMUNO-19 cohort

We set up a national cohort of COVID-19 patients with moderate, severe, or critical pneumonia (CORIMUNO-19 Cohort, NCT04324047). This cohort was used to perform a series of randomized controlled trials testing different therapeutic regimens in COVID-19 patients. Two separate populations were recruited: group 1: patients with moderate-to-severe pneumonia with WHO 10-points Clinical Progression Scale [WHO-CPS] score 5 receiving at least 3L/min O_2_ but without high flow oxygen (HFO), non-invasive ventilation (NIV), or mechanical ventilation (MV) and group 2: patients with critical pneumonia defined as WHO-CPS score 6 or more (i.e., with HFO, NIV or MV) in intensive care unit (ICU).

All patients included in the ancillary study presented in this work were included in the CORIMUNO-19 cohort in three randomized trials evaluating the efficacy of tocilizumab, sarilumab (25) and anakinra (26) in participating hospitals (Hôpital Bicêtre, AP-HP, Université Paris-Saclay; Hôpital Européen Georges Pompidou, Hôpital Bichat, and Hôpital Lariboisière, AP-HP, Université de Paris; Hôpitaux Universitaires de Strasbourg, Université de Strasbourg).

### IFN-α quantification in plasma samples

Plasma and/or serum samples were collected from 84 COVID-19 patients, and 20 healthy donors from a single hospital (Hôpital Bicêtre, Assistance Publique-Hôpitaux de Paris), and 56 COVID-19 patients included in other hospitals participating in the CORIMUNO-19 trials. These samples are part of the French-COVID collection.

Plasma and sera were analyzed using the Simoa technology with the Quanterix SIMOA HD-X analyzer (Quanterix Corporation, Lexington, MA, USA). The levels of plasma IFN-α2 were measured using the commercial kit for IFN-α2 quantifications according to the manufacturer’s instructions (Simoa® IFN-α Advantage Kit, reference 100860; Quanterix). The plasma and serum IFN-α2 levels were expressed in femtogramme per milliliters (fg/mL).

We had both sera and plasma available from 12 additional patients (not included in the analysis since we do not yet have definitive data on the clinical evolution). We could confirm a very good concordance for IFN-α2 for dosages above 100 fg/ml in the plasma. However, the technique was much more sensible with serum for the low levels than with plasma. We could design an algorithm to convert all the plasma values as serum values and analyze the whole cohort of patients together (**Figure S1**).

### Immunophenotyping of peripheral blood leukocytes

Fresh blood samples were used to analyze the phenotype of circulating myeloid cells from COVID-19 patients (n=14) and healthy donors (n=7). Briefly, 200 µL of blood sample was first stained with the Fixable Viability Dye eFluorTM 780 (eBioscience) and then with fluorochrome-conjugated antibodies, including anti-CD45 (BV785; Biolegend, clone 2D1), anti-HLA-DR (APC-R700; BD Biosciences, clone G46-6), anti-CD66b (FITC; Biolegend, clone G10F5), anti-CD3 (FITC; Biolegend, clone SK7), anti-CD56 (FITC; Biolegend, clone NCAM16.2), and anti-CD19 (FITC; Biolegend, clone HIB19), anti-CD14 (BV650; Biolegend, clone M5E2), anti-CD16 (PerCP-Cy5.5; Biolegend, clone 3G8), anti-CD123 (PE; BD Biosciences, clone 7G3), anti-CD11c (BV421; Biolegend, clone 3.9), anti-CD141 (BV510; BD Biosciences, clone 1A4), anti-CD1c (BV711; Biolegend, clone L161). Following incubation with the antibodies, the lysis of red blood cells (RBC) was performed using 1X FACS Lysing Solution (BD Biosciences), and the samples were fixed with 1% PFA. Flow cytometry was performed on a BD LSRFortessa instrument using FACSDiva software (BD Biosciences), and the data were analyzed using FlowJo software (Treestar, San Carlos, CA). DC populations were defined as follows: pDC (CD14*^-^* CD16*^-^* CD123*^+^* CD11c*^-^*), cDC1 (CD14*^-^* CD16*^-^* CD11c^low^, CD141*^+^* CD1c*^+^*), and cDC2 (CD14*^-^* CD16*^-^* CD11c*^+^*, CD141*^-^* CD1c*^+^*). The flow cytometry strategy is indicated in **Figure S2**.

### Functional evaluation of anti-IFN auto-Abs

*Luciferase reporter assays.* The blocking activity of anti-IFN-α2 and anti-IFN-ω auto-Abs was determined with a reporter luciferase activity. Briefly, HEK293T cells were transfected with a plasmid containing the *Firefly* luciferase gene under the control of the human *ISRE* promoter in the pGL4.45 backbone and a plasmid constitutively expressing *Renilla* luciferase for normalization (pRL-SV40). Cells were transfected in the presence of the X-tremeGene9 transfection reagent (Sigma-Aldrich, ref. number 6365779001) for 24 hours. Cells in Dulbecco’s modified Eagle medium (DMEM, Thermo Fisher Scientific) supplemented with 2% fetal calf serum (FCS) and 10% healthy control or patient serum/plasma (after inactivation at 56°C for 20 minutes) were either left unstimulated or were stimulated with IFN-α2 (Milteny Biotec, ref. number 130-108-984), IFN-ω (Merck, ref. number SRP3061), at 10ng/mL or 100pg/mL, or IFN-β (Milteny Biotech, ref. number: 130-107-888) at 10ng/mL, for 16 hours at 37°C. Each sample was tested once for each cytokine and dose. Finally, cells were lysed for 20 minutes at room temperature, and luciferase levels were measured with the Dual-Luciferase® Reporter 1000 assay system (Promega, ref. number E1980), according to the manufacturer’s protocol. Luminescence intensity was measured with a VICTOR-X Multilabel Plate Reader (PerkinElmer Life Sciences, USA). *Firefly* luciferase activity values were normalized against *Renilla* luciferase activity values. These values were then normalized against the median induction level for non-neutralizing samples and expressed as a percentage. Samples were considered neutralizing if luciferase induction, normalized against *Renilla* luciferase activity, was below 15% of the median values for controls tested the same day.

### Plasma viral load

As previously published plasma samples were extracted using the Cellfree200 V7 DSP 200 protocol with the QIAsymphony® DSP virus/pathogen mini kit (QIAGEN, UK) (27). Samples loaded onto the QIAsymphony® SP as instructed by the manufacturer, with a 200 μl sample input volume (composed of 150µl of plasma and 50µl of NaCl) and 60 μl elution output volume of AVE buffer, unless stated (QIAGEN, UK). The samples were then stored at -80°C until the droplet digital PCR (ddPCR) step. SARS-CoV-2 RT-ddPCR assays were performed using the One-Step RT-ddPCR Advanced Kit for 90 Probes (Bio-Rad Laboratories, Hercules, CA, USA) and the QX200 ddPCR platform (Biorad). A 2-plex RT-ddPCR assay was developed, which targets the Nucleocapsid (N1) gene of the SARS-CoV-2 positive-strand RNA genome with a specific FAM- probe, and primers Cy5-labeled probe for the detection of a human housekeeping gene (RNAseP). RNAseP positivity was necessary to validate the RT-PCR assay prior to any further analysis. Briefly, 9.9 μL of extracted RNA was diluted in a 22 μL final reaction volume containing 5.5 μL of One Step SuperMix, 2.2 μL of Reverse Transcriptase, 1.1 μL of 300mM DTT (One-Step RT-ddPCR Advanced Kit for Probes, Bio-Rad), 1.1 μL of primers and probes mix (final probe concentration: 200 nM each, final primer concentration: 600 nM each) and 2.2 µL (QS) of nuclease-free water. Then, each sample was primarily partitioned into 13000 to 20,000 droplets using the QX200™ Droplet Generator™ (Bio-Rad). PCR amplification was then performed on a C1000 Touch™ thermal cycler (Bio-Rad). The droplet reading and quantification were performed using the QX200™ Droplet Reader™, and data analysis was performed using the QuantaSoft™ Analysis Pro software (Bio-Rad). Plasma samples were considered of positive SARS-CoV2 RNA if RNAseP concentration was detected above 3.4 log copies per mL and SARS-CoV-2 RNA concentration above the assay limit of detection of 70 copies per mL.

### Statistical analysis

Kruskall Wallis test, followed by a Dunn’s test, was used to compare unpaired multiple groups and determine p values. For paired data comparison of several groups, one-way ANOVA was performed, followed by Dunnett’s post-test to determine the p values between groups. For paired data comparison of two groups (severe vs. critical patients), divided into subgroups (D1, D3, D6, D14), two-way ANOVA was used, followed by Sidak’s post-test to determine p values in between each subgroup (comparison of the IFN-α level between the different time points). For paired data comparison of two groups (severe vs. critical) divided into subgroups (D1, D3, D6, D14), a two-way ANOVA test was used, followed by Tukey’s post-test to determine p values between the different time points compared to D1). Because the longitudinal serum IFN-α2 levels and the process of death could be interrelated, we used a joint model for the longitudinal process of IFN-α2 (after log transform) and survival between D1 and D6 was used to estimate individual slopes of variation of IFN-α2 levels between D1 and D6 while accounting for the informative censoring due to death and the association of IFN-α2 level with the death process. In this model, given the small number of longitudinal measurements between D1 and 6, a linear mixed effects model was used for the longitudinal process, with random intercept and slope. A proportional hazards model was assumed for the survival process for the time-specific IFN-α2 values, and the baseline hazard was approximated with splines. The model was fitted under a Bayesian approach using the JMBayes R package (28), accounting for the quantification limit of IFN-α2 (5 fg/ml in the serum and 96.37 fg/ml in the plasma). Slopes were then dichotomized at the median value to separate the half patients with the steepest IFN-α2 relative decrease between D1 and D6 (slope < median) and the others (slope > median). A landmark survival analysis with a landmark at 6 days was then used to compare the survival of both groups. This entails analyzing individuals alive at D6 only but avoids time-dependent bias. Statistical analyses were performed with R 3.6.3 (The R Foundation for Statistical Computing, Vienna, Austria) and GraphPad Prism.

### Study approval

All methods were performed in accordance with the relevant guidelines and regulations. The trial was approved nationally by the ethics committee on March 23, 2020 (file #20.03.20.56342, CPP Île De France VI, EudraCT: 2020-001246-18), by the French Medical Products Agency and by the Commission Nationale Informatique et Liberté. Written informed consent was obtained from all patients or from the patient’s legal representative if the patient was too unwell to consent to enter the CORIMUNO Cohort.

## Results

### Baseline levels of plasma IFN-α2, anti-IFN-α auto-Abs, and dendritic cells in patients with severe and critical COVID-19

To better understand the role of type I IFN in the evolution of COVID-19, we performed a longitudinal study of blood IFN-α2 levels in 140 patients from the CORIMUNO-19 cohort (NCT04324047) divided into two groups, group 1: patients with severe COVID-19 requiring at least 3L/min of oxygen (n=81) and group 2: patients with critical COVID-19, requiring high-flow, non-invasive (NIV) or mechanical ventilation (MV) in intensive care unit (ICU) (n=59) (25). Twenty healthy blood donors were used as controls. The patients’ and controls’ baseline characteristics are indicated in **Table 1**.

**Table 1:** Baseline characteristics of the patients.

	Healthy donors (n=20)	Severe patients (n=81)	Critical patients (n=59)	Total patients (n=140)
Age (years)	41[27-55]*	62 [52-70]	61 [54-66]	61 [54-70]
Male, n/N (%)	12/17 (71%)*	53/81 (65%)	47/59 (80%)	100/140 (71%)
Time from symptoms onset to randomization (days)		10 [7-12], n=80	11 [8-14], n=58	10 [8-13], n=138
WHO score (0-10)				
5		81/81 (100%)	0	81/140 (58%)
6 (NIV)		0	16/59 (27%)	16/140 (11%)
7–9 (MV)		0	43/59 (73%)	43/140 (31%)
Flow (L/min)		5 [4-6]	40 [34-60], n=16	5 [4-9], n=97
Neutralizing IFN-α2 auto-Abs, n (%)		3 (3.7%)	3 (5.2%)	6 (4.3%)
Neutrophils (G/L)		6.0 [4.3-7.6]	6.7 [4.9-9.5], n=56	6.4 [4.6-8.1], n=137
Lymphocytes (G/L)		0.9 [0.7-1.2]	0.9 [0.6-1.1], n=56	0.9 [0.6-1.2], n=137
Lymphocytes to neutrophils ratio		0.16 [0.11-0.25]	0.12 [0.08-0.16], n=56	0.14 [0.10-0.22], n=137
D Dimers (µg/L)		1061 [775-1695], n=74	1836 [1200-3126], n=50	1378 [845-2070], n=124
Evolution to death, n (%)		14 (17%)	15 (25%)	29 (21%)

*For the 20 healthy donors, data are unavailable only for 17 of them.

Values are median [interquartile range] unless stated otherwise.

**Figure 1:**
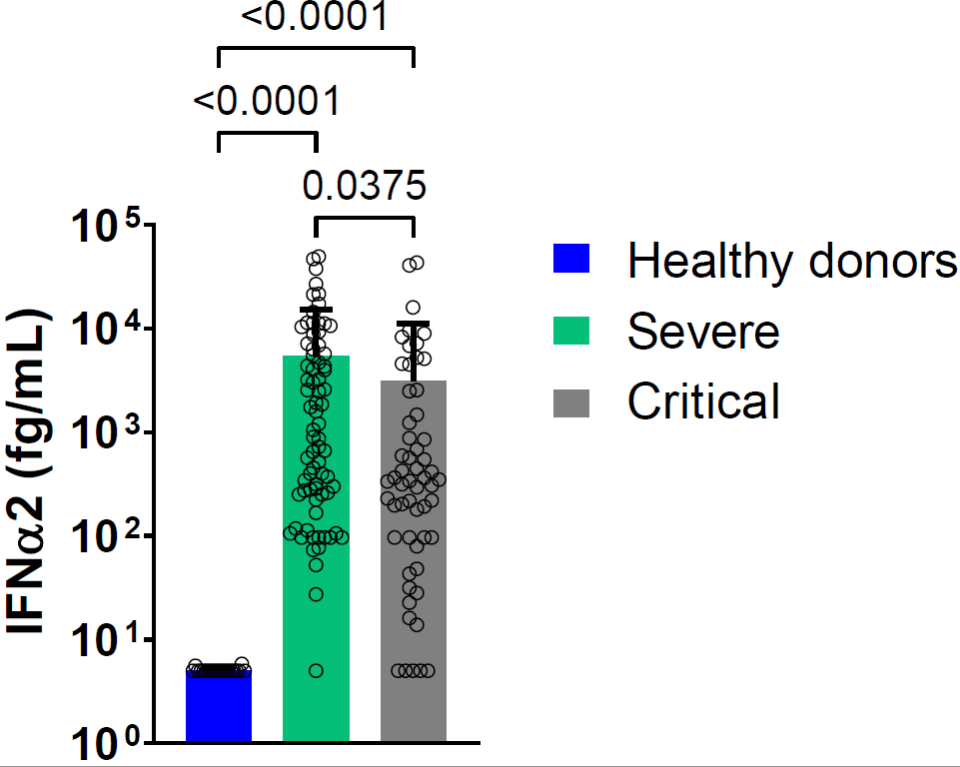
Mean blood IFN-α2 level at D1 in severe and critical COVID-19 patients and in healthy donors. Bars represent the mean level of IFN-α2 in femtogram per milliliter (fg/mL) for each group: severe patients (green bar, n_serum_ =23, n_plasma_=52), critical patients (light grey bar n_serum_ =31, n_plasma_=26), healthy donors (blue bar, n=20). Symbols represent individual measures done at D1. Detection of IFN-α2 in serum and plasma pooled. Statistical analysis was performed using Kruskal Wallis’s test, followed by Dunn’s post-test.

The baseline (at entry in the hospital [D1]) blood level of IFN-α2 was elevated in both groups compared to controls with higher levels in severe patients compared to critical patients (**Figure 1**). The results remain the same if we consider separately dosages made on serum and plasma (**Figure S3**). Baseline and day 6 blood IFN-α2 levels were not associated with survival (**Figure S4**).

The anti-IFN-α2 auto-Abs detected were neutralizing against 10 ng/mL IFN-α2 in 3.7% and 5.2% of severe and critical patients, respectively, and against 100 pg/mL IFN-α2 in 6.2% and 5.2%, of severe and critical patients, respectively. The presence of neutralizing anti-IFN-α2 auto-Abs to both doses of IFN-α2 was associated with a lower level of blood IFN-α2 at baseline (**Figure S5A**). Interestingly, in these patients, the blood IFN-α2 levels were persistently low, although not associated with subsequent mortality either in severe or critical patients (**Figure S5B**). If we consider anti-IFN-α2 or anti-IFN-ω, neutralizing anti-IFN auto-Abs were present at high and low concentrations (10 ng/mL and 100 pg/ml) in 5% and 19.4% of patients, respectively, but with no correlation with the level of blood IFN-α2 and with subsequent mortality.

**Figure 2:**
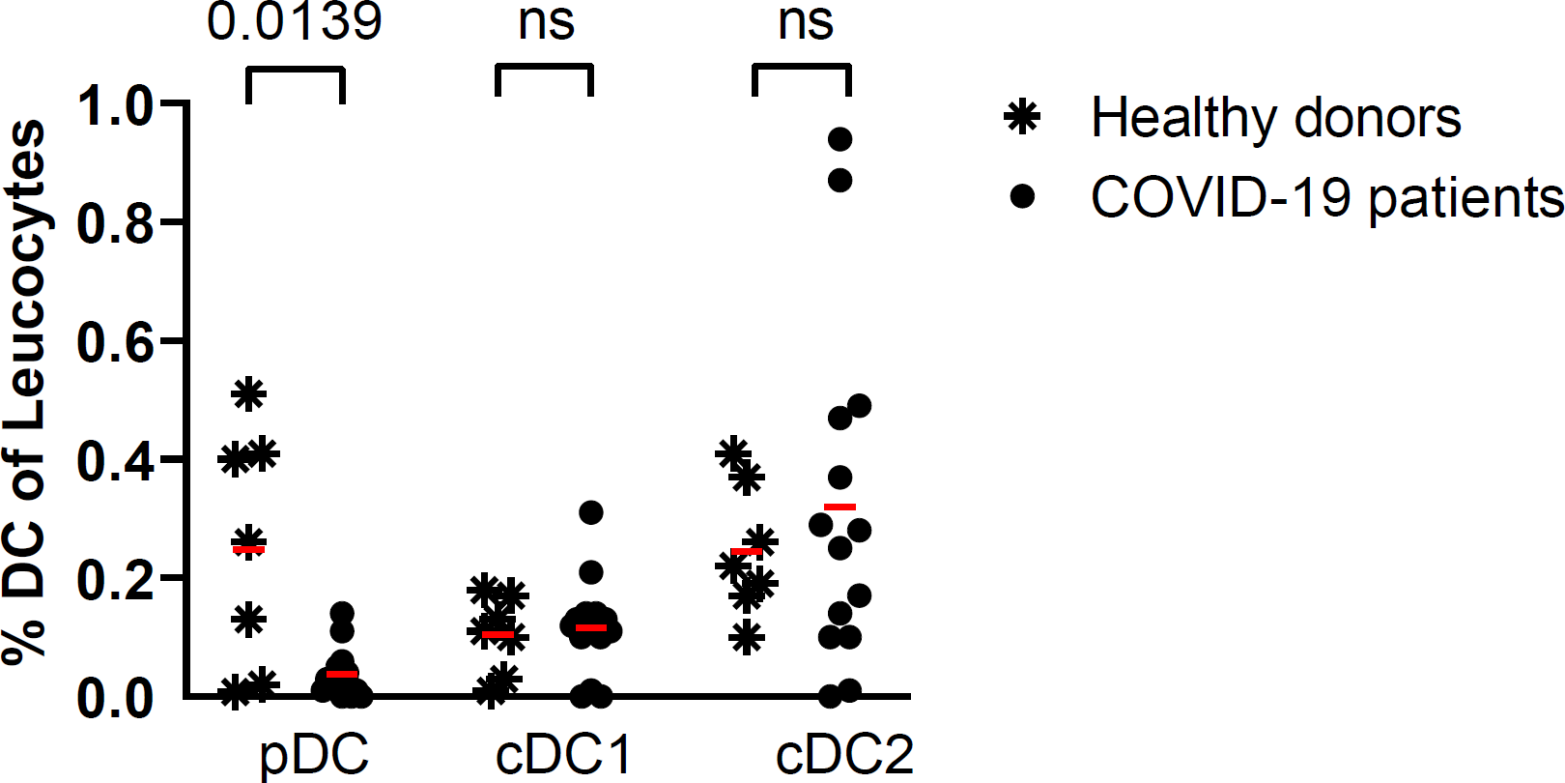
Percentage of DC subsets in COVID-19 patients (n=14) at baseline and in healthy donors (n=7). Red lines correspond to the mean percentages of DC (percentage of leucocytes) in both groups; symbols represent individual values (asterisk for healthy donors and circle for COVID-19 patients) (Kruskal Wallis’s test, followed by Dunn’s post-test). ns = non significant.

In the peripheral blood of a subset of patients (n=14), compared with healthy donors (n=7), the percentage of pDC was low. In contrast, the percentage of conventional dendritic cells (cDC1) and cDC2 was normal (**Figure 2**). A low percentage of peripheral blood pDC was present in most of these patients with severe or critical COVID-19 pneumonia. This decrease in pDC was not correlated with the baseline, D6, or the D6/D1 ratio of plasma IFN-α2 level. The baseline percentage of pDC was not predictive of subsequent mortality.

### Longitudinal follow-up of plasma IFN-α2 in relation to mortality in patients with severe and critical COVID-19

**Figure 3:**
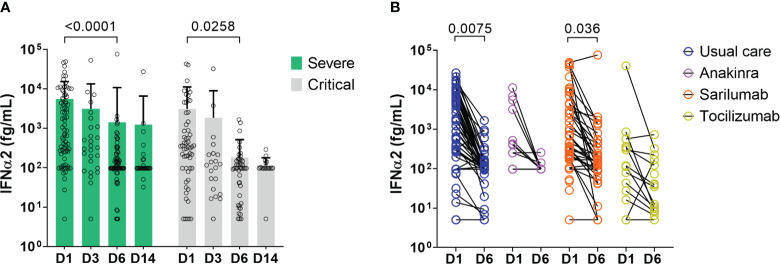
Change in blood IFN-α2 over time in severe and critical patients. **(A)** Results of blood IFN-α2 dosage at D1, D3, D6, and D14. Bars represent the mean level of IFN-α2 in fg/m for each group: severe patients (green bar) and critical patients (light grey bar), and symbols represent individual measures. Blood IFN-α2 waned between D1 and D6 in severe and critical patients (Two-way ANOVA, followed by Sidak’s post-test). **(B)** Blood IFN-α2 level at D1 and D6 in COVID-19 patients treated with anakinra (n=12), sarilumab (n=49), tocilizumab (n=17), or usual care (n=62). Symbols represent individual measures. Patients were separated into 4 groups according to the treatment: anakinra (purple symbols), sarilumab (orange symbols), tocilizumab (yellow symbols), or usual care (dark blue symbols). The black lines link D1 and D6 IFN-α2 symbols to visualize the evolution between the two time points for each patient. Only patients with available dosage at D1 and D6 are represented (patients dead in between those days or without available serum at D6 are depicted). IFN-α2 levels of patients treated with usual care, anakinra, and tocilizumab waned between D1 and D6 (Two-way ANOVA, followed by Sidak’s post-test).

**Figure 4:**
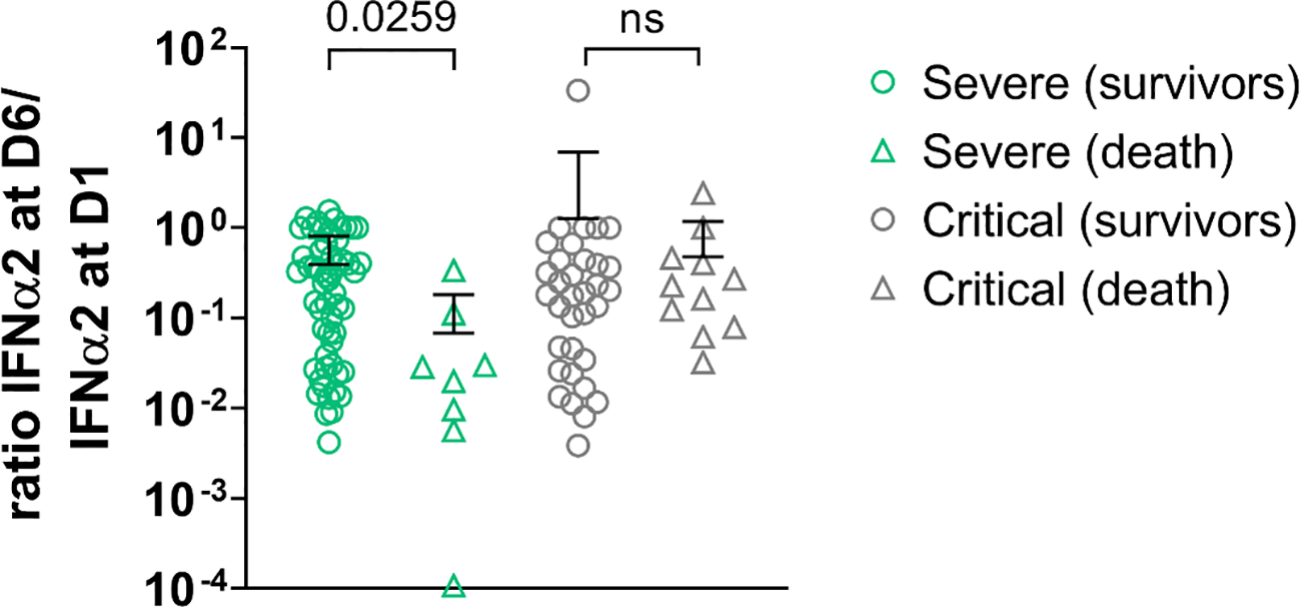
D6/D1 ratio of IFN-α2 level in severe patients and critical patients. Symbols represent individual measures: severe patients (green symbols, n=64) and critical patients (light grey symbols, n= 45). The lines represent the mean ratios; patients who died are represented with triangles (n=19) and survived patients in circles (n=90). Among the severe patients, those who perished depicted lower ratios IFN-α2 D6/IFN-α2 D1 than those who survived. There was no difference in ratios among critical patients (Kruskal Wallis’s test, followed by Dunn’s post-test). ns = non significant.

In order to determine whether IFN-α2 variation during disease evolution may have a predictive value, we collected samples over time to assess blood IFN-α2. Blood IFN-α2 levels decreased in both groups of COVID-19 patients over time (**Figures 3A**, **S6**) and did so regardless of the treatment group (usual care (UC), sarilumab, tocilizumab, or anakinra) (**Figure 3B**). Baseline (D1) IFN-α2 was not different between groups and treatment did not induce different changes in IFN-α2 between groups (data not shown). Thus, we merged the treatments group and analyzed the severe and critical groups. The magnitude of the decrease measured by the D6/D1 blood IFN-α2 ratio was significantly higher in severe patients who subsequently died than in patients who survived (p=0.026) (**Figure 4**). Separated dosages on serum and plasma are shown in **Figure S7**. Interestingly, the D6/D1 blood IFN-α2 ratio was predictive of subsequent mortality only in patients without neutralizing anti-IFN-α2 auto-Abs (data not shown), probably because IFN-α2 levels were already low at baseline in patients with neutralizing anti-IFN-α2 auto-Abs (**Figure S5**).

### A statistical mixed model confirmed the link between the slope of the decrease of blood IFN and subsequent death

**Figure 5:**
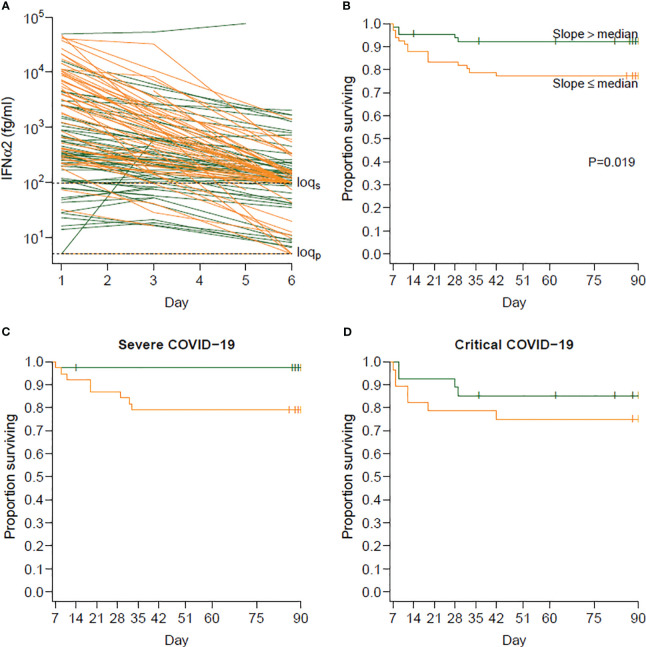
Relationship between the slope of IFN-α2 between days 1 and 6 and survival beyond day 6. **(A)** Observed evolution of IFN-α2 according to whether the individual slopes of variation of IFN-α2 was below (orange, steeper decrease, n=66) or above (green, slower decrease, n=65) the median slope. The slopes were obtained by modeling the log-transformed IFN-α2 values in a Bayesian joint model accounting for limits of quantification and death. **(B)** Survival beyond day 1 in day 6 survivors (landmark analysis) according to the same groups of individuals. Individuals with a steeper decrease in IFN-α2 (in orange) had a higher risk of death than those with a slower decrease (in green) (hazard ratio [HR] 3.15; 95% confidence interval [CI] 1.14–8.66; p=0.019). **(C)** Survival beyond day 1 in day 6 survivors for severe patients at baseline. **(D)** Survival beyond day 1 in day 6 survivors for critical patients at baseline.

Since computing the D6/D1 ratio is complicated by values under the limit of quantification (LOQ) and because the longitudinal serum IFN-α2 levels and the process of death could be interrelated, we used a joint model for the log IFN-α2 levels and death during the first 6 days in order to estimate a slope of log IFN-α2 for each individual, thereby providing similar information as the D6/D1 ratio (**Figure 5A**). Using a landmark analysis and dichotomizing the individual slopes at their median value, we then found that the half of patients with the steepest slope (lowest D6/D1, n=66) had higher mortality than the others (n=65): HR=3.15 (95% CI 1.14–8.66) in rapid decliners versus slow decliners (p=0.019) (**Figure 5B**), without interaction between severe and critical patients (p=0.21) (**Figures 5C, D**).

### Baseline and longitudinal follow-up of plasmatic SARS-CoV-2viral load

**Figure 6:**
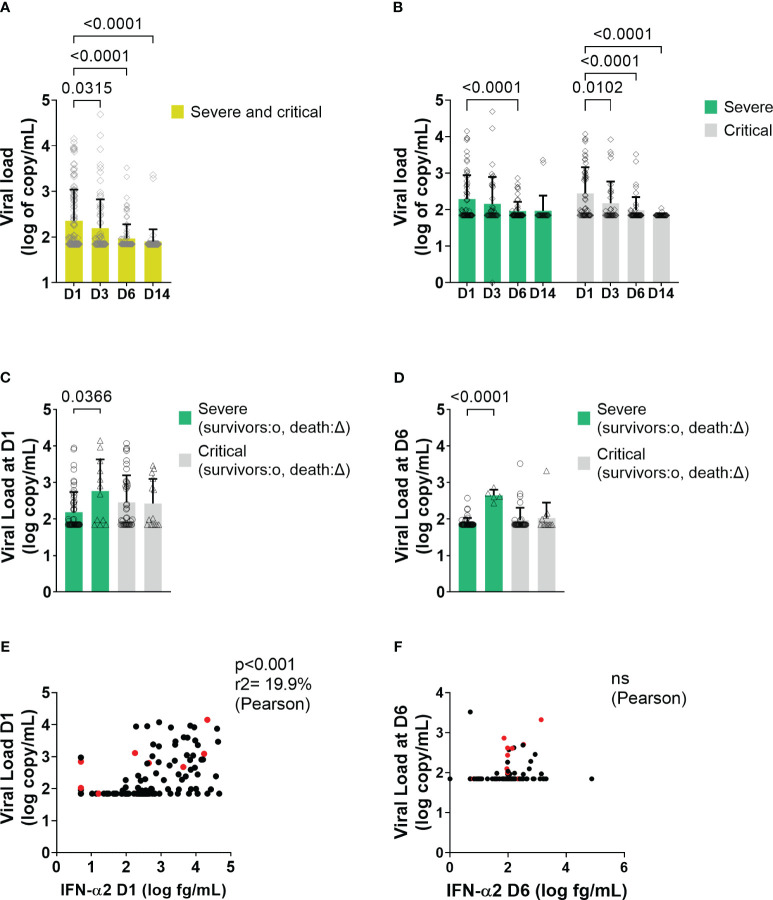
Viral load and correlation with IFN-α2 in all patients and in severe and critical patients. **(A, B)** Mean level of plasma viral load level at different time points in COVID-19 patients. Bars represent the mean level of plasma viral load in log_10_ of copy/mL of all patients. Symbols represent individual measures. Plasma viral loads waned from D1 to D3, D6, and D14 of the follow-up (One-way ANOVA followed by Dunnett’s post-test comparing all time points to D1). **(C, D)** Mean level of plasma viral load between survivors and dead in severe and critical patients at D1 and at D6, respectively (*Two-way ANOVA followed by Tukey’s post-test*). **(E, F)** Correlation between viral load and blood IFN level at D1 and at D6, respectively. On the X-axis are depicted the IFN-α2 a values in log_10_ (fg/mL), and on the Y-axis, the viral load values expressed in log_10_ (copy/mL) (Pearson’s correlation test). Red points correspond to patients who will not survive. ns = non significant.

Viral load was detectable in plasma at baseline (mean 10 days from the first symptoms) in 70/140 (50%) patients and significantly decreased progressively over time in both groups (**Figures 6A, B**). Interestingly, severe patients who died after day 6 displayed a higher viral load at D1 and D6 than patients who survived (**Figures 6C, D**). SARS-CoV-2 viral load and serum IFN-α2 level correlated at admission (D1) (**Figure 6E**) but not at D6 (**Figure 6F**).

## Discussion

In this prospective study, using the Simoa technology, we confirmed that type I IFN response was elevated in patients with severe and critical COVID-19 pneumonia, compared with healthy controls (14–17, 19, 20, 29) correlated with plasma viral load, and associated with a decrease in pDC in blood, perhaps due to migration of these cells into the lung, the active site of the viral infection, as demonstrated for HIV infection (30, 31). Indeed, pDCs were recently shown to be essential for the control of SARS-CoV-2 in the lungs (17). Furthermore, we found that a rapid decline of blood IFN-α2 was associated with subsequent mortality using two different statistical methods.

The question persists in determining if the level or kinetic of type I and III IFN response is predictive of the worst outcome (13). Regarding type I IFN, two papers concerning the same cohort of patients recruited at Yale University suggested that IFN-α levels, elevated at baseline, were sustained in the course of the disease in patients with severe illness but declined in patients with moderate disease (20, 32). Conversely, Trouillet-Assant et al. and Hadjadj et al. have found a profoundly impaired type I IFN signature in critically ill ICU patients compared with less severe patients, accompanied by hyperinflammatory status (14, 15). Considerable evidence suggests that a defect of type I IFN biological effect through impaired secretion or signaling could be deleterious for COVID-19 outcome. In particular, studies suggest that the impossibility of maintaining an increased or effective type I IFN response is associated with the worst outcome. The first ones showed that rare inborn errors of Toll-like receptor-7 (TLR-7) (33, 34), or TLR3- and interferon regulatory factor 7 (IRF7)-dependent immunity genes were associated with life-threatening COVID-19 pneumonia (16). More recently, a larger study reported that 1.5% of male patients with critical COVID-19 pneumonia had X-recessive TLR7 deficiency, incriminating pDCs in the pathogenesis (17). Defect in type I IFN action could also be caused by pre-existing neutralizing auto-antibodies against type I IFN that were measured in at least 10% of critically ill COVID-19 patients, mainly older ones (29). These findings have been replicated in five other cohorts (18, 35–39) and recently in a very large sample that also reported pre-existing neutralizing antibodies against lower concentrations (100 pg/mL) of IFN-α2 in 20% of COVID-19 deaths and 20% of critical cases in patients >80 years (19). Thus, anti-IFN neutralizing antibodies are much more prevalent than variants of immunity genes with loss of function. In our study, neutralizing anti-IFN-α2 auto-Abs at 10 ng/mL were detected in around 5% of the severe and critical patients (3.7% and 5.2% respectively), in line with the age distribution of the CORIMUNO-19 sample (average 61 years [54–70]). We could not find a statistical association of the presence of neutralizing anti-IFN-α2 auto-antibodies and the risk of death. This might be because all enrolled patients were at least severely ill, and most of the literature data concerning anti-IFN-α2 auto-Abs showed a difference in prevalence between asymptomatic/mild versus severe forms (16, 17, 29). Another explanation is a potential lack of statistical power, as autoantibodies prevalence accounted for only 5% of the cohort. Following previous studies, the presence of anti-IFN-α2 auto-Abs was associated with a lower baseline blood IFN-α2 level. It has been well demonstrated by JL Casanova’s group that the presence of anti-IFN-α2 auto-Abs neutralized IFN *in vitro* (19, 29), and thus, the low level of blood IFN in these patients is not due to our assay. Interestingly, the change in blood IFN-α2 levels as assessed by the D6/D1 blood IFN-α2 ratio was predictive of death only in patients with no anti-IFN-α2 auto-antibodies.

Only one previous study provided longitudinal follow-up of type I IFN measurement in patients with COVID-19 (40). This study included only 8 patients with severe or critical COVID. Like in our study, in comparison to less severe patients, these patients tested at entry in hospital 8 days after symptoms onset had a higher level of serum INF-α level that stepped down over time. Interestingly, in this study, INF-α and viral load measured on swab samples had a trend to correlate, correlation that we have shown at baseline between serum INF-α2 level and viral load measured on plasma. However, none of these patients died and thus, it was not possible to correlate the slope of decrease of INF-α and subsequent death. Therefore, in order to draw conclusion on the link between IFN-α kinetics and prognosis, it was mandatory to set up a prospective longitudinal study including more patients with different time points of type I IFN dosage associated with a clinical follow-up. Thanks to the prospective follow-up, we bring strong arguments supporting the fact that, in seronegative patients, it is indeed the decline of IFN-α2 and not its baseline level that predicts higher severity and mortality.

IFN-α2 circulating levels declined in all patients. Nevertheless, the link between this decline and subsequent mortality was statistically significant in severe but not in critical patients. It might be due to a question of statistical power since the number of critical patients was lower. It may also be linked to the fact that the decline in type I IFN plays a crucial role in severe patients, but once the patients are mechanically ventilated in ICU, other factors are at least as important for predicting death.

The recent development of ultrasensitive PCR technology for absolute quantification of nucleic acids in plasma, such as droplet-based digital PCR (ddPCR), could show that the prevalence of positive SARS-CoV-2 RNAaemia correlated with disease severity in subjects with mild-to-moderate to severe or critical COVID-19 disease (23). An independent relationship of diabetes with a higher SARS-CoV-2 RNAemia in critically ill COVID-19 patients was also reported, and in multivariable logistic regression models, SARS-CoV-2 RNAemia was strongly and independently associated with day-60 mortality (27). In the present study, using the same sensitive and specific assay, we further found that SARS-CoV-2 RNAaemia at D1 and D6 was associated with death risk in this larger cohort of unselected severe patients needing oxygen support. Another original finding is that SARS-CoV-2 viral load and serum IFN-α2 level correlated at admission, on average after 10 days from symptoms onset, suggesting a relationship between systemic viral load and IFN response that was lost afterward.

Our study has some limitations. First, the treatment of these patients was heterogeneous in the frame of randomized trials; however, it is noteworthy that the decrease of type I IFN over time was observed regardless of the treatment group. Moreover, none of the used drugs is known to interfere with the type I IFN pathway; and the treatment had no impact on the IFNα2 D1/D6 ratio. Last, we could have hypothesized that the most severe patients could have received a higher dose of steroids that could have decreased IFNα2 level. It was not the case since our trial was conducted before the demonstration of efficacy of steroids in severe and critical COVID-19: only 20% and 13% of patients had received steroids in the CORIMUNO sarilumab and anakinra trials, respectively (25, 26) and almost none of them received dexamethasone. Second, we focused on the sole IFN-α2 isotype only; third, we provided data from blood and did not demonstrate a decrease in type I IFN in the lung. Interestingly, a decreased type I IFN signature has been described in bronchoalveolar lavage macrophages from severe COVID-19 patients (41), supporting that what we observed in blood also probably occurred in the lungs.

Our study could support the interest of using Type I IFN as a treatment for patients with severe COVID-19. A recent randomized controlled study demonstrated the efficacy of pegylated interferon lambda (type III interferon) for preventing severe outcome in patients with early acute COVID-19 (3 to 7 days after the beginning of symptoms) and one risk factor of severe COVID-19 (42). In a randomized controlled study having included 250 patients, early treatment with PEG IFN- α2b induced early viral clearance and improved the clinical status of patients with moderate COVID- 19. It also decreased the duration of supplemental oxygen (43). In another uncontrolled exploratory study in 77 patients with moderate COVID-19, treatment with nebulized IFN-a2b significantly reduced the duration of detectable virus in the upper respiratory tract and in parallel reduced duration of elevated blood levels for the inflammatory markers IL-6 and CRP (44). Conversely to the concern about a possible pro-inflammatory effect of IFN-a2b (45), this study showed, conversely, a decrease of inflammatory markers in patients treated with nebulized IFN-a2b (44). Thus, based on our results, interferon treatment could also be considered in association with dexamethasone +/- tocilizumab in severe patients with rapid IFNa2 decline.

In conclusion, these data suggest that patients with severe COVID-19 pneumonia have an increased type I IFN level in relation to the viral load. Patients who are worsening do not maintain a sufficiently high level of type I IFN. Reciprocally, SARS-CoV-2 RNAaemia at D1 and D6 was associated with death risk. The mechanisms of this decrease of type I IFN in the most severe patients over time have to be elicited. It is the condition for discussing the possible interest of evaluating type 1 IFN treatment in patients with severe COVID-19 who harbor type 1 IFN decline before the critical phase of the disease, eventually combined with anti-inflammatory drugs like dexamethasone and IL-6 inhibitors.

## Data availability statement

The original contributions presented in the study are included in the article/**Supplementary material**. Further inquiries can be directed to the corresponding author.

## Ethics statement

The studies involving humans were approved by CPP Île De France VI on March 23, 2020 (file #20.03.20.56342). The studies were conducted in accordance with the local legislation and institutional requirements. The participants provided their written informed consent to participate in this study.

## Author contributions

Biomonitoring and biobanking: CV, A-MR-A. Design, biomonitoring, and experiment (Simoa): CJ, DD, RLG. Design, biomonitoring, and experiment (viral load): P-LT, HP, TB, LR, OL, DV. Design and experiment (autoantibodies): AC, PB, QZ, J-LC. Statistical analysis and data representation: CL, CJ, DD, RP, GB. Interpretation of the results: CJ, DD, RP, XM. Clinical trial coordination and design: XM, LS, OH, MR-R, PR. Manuscript writing (original draft): XM. Writing, Review, and editing: P-LT, HP, OH, CV, PB, J-LC, DD, CJ, RLG.
