# Supplementary material for: More rapid blood interferon α2 decline in fatal versus surviving COVID-19 patients
Source: Front Immunol. 2023 Nov 21;14:1250214. doi: 10.3389/fimmu.2023.1250214 (PMC10703045; doi:10.3389/fimmu.2023.1250214)
Supplement: Supplementary file 1 [file DataSheet_1.docx]

**Acknowledgments**

**CORIMUNO-19 steering committee**

Philippe Ravaud (chair of the CORIMUNO-19 platform), Serge Bureau, Maxime Dougados, Olivier Hermine, Xavier Mariette, Matthieu Resche-Rigon, Pierre-Louis Tharaux, Annick Tibi

**CORIMUNO-19 scientific committee**

Olivier Hermine (chair), Elie Azoulay, Serge Bureau, Jacques Cadranel, Maxime Dougados, Joseph Emmerich, Muriel Fartoukh, Bertrand Guidet, Marc Humbert, Karine Lacombe, Matthieu Mahevas, Xavier Mariette, Frédéric Pene, Raphaël Porcher, Valérie Pourchet-Martinez, Philippe Ravaud, Matthieu Resche-Rigon, Frédéric Schlemmer, Pierre-Louis Tharaux, Annick Tibi, Yazdan Yazdanpanah

**CORIMUNO-19 clinicians and participants from Bicêtre hospital**

***Investigators***: Xavier Mariette, Laurent Savale, Anatole Harrois, Samy Figuereido, Jacques Duranteau, Nadia Anguel, Xavier Monnet, Christian Richard, Jean-Louis Teboul, Arthur Pavot, Philippe Durand, Pierre Tissieres, Mitja Jevnikar, David Montani, Marc Humbert, Stephan Pavy, Samuel Bitoun, Julien Henry, Nicolas Noel, Olivier Lambotte, Christelle Chantalat-Auger, Lelia Escaut, Stephane Jauréguiberry, Elodie Baudry, Christiane Verny, Edouard Lefevre, Mohamad Zaidan

***Local Clinical Research unit***: Domitille Molinari, Gaël Leprun, Alain Fourreau, Laurent Cylly, Lamiae Grimaldi

***Local Clinical Research team***: Myriam Virlouvet, Ramdane Meftali, Solène Fabre, Marion Licois, Asmaa Mamoune

***Pharmacy:*** Clotilde Le Tiec

***Biological Resource Center***: Céline Verstuyft, Anne-marie Roques, Julie Tisserand, Nisrine Soltani, Allison Cardoso, Ines Martins

***Clinicians having taken care of patients*:** Laurent Guerin, Astrid Bertier, Soufia Ayed, Thibault Creutin, Tai Pham, Frédéric Desmoulins, Matthieu Guillet, Manon Dekeyser, Olivier Sitbon, Etienne Marie Jutant, Xavier Jais, Jérémie Pichon, Athenais Boucly, Sophie Bulifon, Andrei Seferian, Antoine Beurnier, Florence Parent, Roseline D’Oiron, Antoine Cheret, Jeremy Gottlieb, Jérémie Benichou, Alexandre Dormoy, Céline Labeyrie, Julien Henry, Alexandre Virone

***Clinical Research technicians, clinical research doctors* and students:** Myriam Virlouvet & Solène Fabre (Gynaecology-obstetrics dpt), Yacine Boudali, Sylvie Miconnet (Rheumatology dpt), Marie-Thérèse Legaud (Nephrology dpt), Jugurtha Berkenou (Plateforme Maladies Rares), Anne Brel, Andreea Catina, Céline Chevreau, Jodie Ferrand, Marie Galopeau, Noémie Monard, Clémence Roche, Cypria Siva, Béatrice Vest, Kévin Hakkakian, Peter Chen, Lucile Durand, Jeremie Robinsohn, Yohann Cauche, Simon Rigaux, Léa Delarue, Claire Carles, Céline Koeberle, Baptiste Desjardins, Thomas Boesch, Nicolas Leroux, Pauline Potisek, Christian Luna, Khalis Sarah, Chabbert Jerôme, Bourdin Joachim, Lola Othily, Constans Fanny, Rosalie Brabant, Nassim Benaissa, Alexandre Khuu, Ariane Guillaume, Neslon Droguete Gomes, Djilali Batouche, Clémence Mille, Camille Hostachy Arnaud Peramo, Marc Labetoulle, Antoine Rousseau

**UMR1184/IMVA-HB participants**

Marie Bitu, Christine Bourgeois, Anaelle Olivo, Juliette Pascaud, Bineta Ly, Florence Abdallah, Julien Lemaitre, Benoit Favier, Olivier Lambotte

**CORIMUNO-19 clinicians and participants from Georges Pompidou European hospital**

***Investigators***: Jean-Benoit Arlet, Jean-Luc Diehl, Florence Bellenfant, Anne Blanchard, Alexandre Buffet, Bernard Cholley, Antoine Fayol, Edouard Flamarion, Anne Godier, Thomas Gorget, Sophie-Rym Hamada, Caroline Hauw-Berlemont, Jean- Sébastien Hulot,David Lebeaux, Marine Livrozet, Adrien Michon, Arthur Neuschwander, Marie-Aude Penet, Benjamin Planquette, Brigitte Ranque, Olivier Sanchez, Geoffroy Volle, Stéphanie Baron.

***Local Clinical Research unit*** : Sandrine Briois, Mathias Cornic, Virginie Elisee, Denis Jesuthasan, Juliette Djadi-Prat, Pauline Jouany, Ramon Junquera, Mickael Henriques, Amina Kebir, Isabelle Lehir, Jeanne Meunier, Florence Patin, Valérie Paquet, Anne TréHan, Véronique Vigna.

***Pharmacy:*** Brigitte Sabatier.

***Biological Resource Center and Inserm UMR970 lab*** : Damien Bergerot, Charlène Jouve, Camille Knosp, Olivia Lenoir, Léa Resmini, Stéphanie Baron, Nassim Mahtal.

**CORIMUNO-19 clinicians and participants from Hôpitaux Universitaires de Strasbourg, Université de Strasbourg**

***Investigators:*** Jacques-Eric Gottenberg, Yves Hansmann Frédéric Blanc, Sophie Ohlmann-Caillard, Vincent Castelain, Emmanuel Chatelus, Eva Chatron, Olivier Collange, François Danion, Frédéric De Blay, Pierre Diemunsch, Sophie Diemunsch, Renaud Felten, Bernard Goichot, Valentin Greigert, Aurelien Guffroy, Bob Heger, Charlotte Kaeuffer, Loic Kassegne, Anne Sophie Korganow, Pierrick Le Borgne, Nicolas Lefebvre, Paul-Michel Mertes, Eric Noll, Mathieu Oberlin, Vincent Poindron, Julien Pottecher, Yvon Ruch, François Weill

***Local Clinical Research unit:*** Nicolas Meyer, Emmanuel Andres, Eric Demonsant, Hakim Tayebi, Gabriel Nisand, Stéphane Brin, Cédric Sublon

***Pharmacy:*** Guillaume Becker, Anne Hutt, Tristan Martin

***Biological Resource Center***: Sophie Bayer, Catherine Metzger

**AP-HP, Hôpital Lariboisière, Université de Paris**

***Investigators*** : Damien Sène, Ruxandra Burlacu, Benjamin Chousterman, Bruno Mégarbanne, Pascal Richette, Jean-Pierre Riveline, Aline Frazier

***Local Clinical Research unit*** : Eric Vicaut, Laure Berton, Tassadit Hadjam, Miguel Alejandro Vazquez-Ibarra, Clément Jourdaine, Olivia Tran, Véronique Jouis

***Pharmacy*** : Aude Jacob, Julie Smati, Stéphane Renaud

***Biological Resource Center***: Claire Pernin, Lydia Suarez

**AP-HP, Hôpital Bichat, Université de Paris**

***Investigators***: Xavier Lescure, Jade Ghosn, Antoine Bachelard, Anne Rachline, Valentina Isernia, Bao-chau, Phung, Dorothée Vallois, Aurelie Sautereau, Catherine Neukrich,  Antoine Dossier,  Raphaël Borie,  Bruno Crestani, Gregory Ducrocq Philippe Gabriel Steg, Philippe Dieude, Thomas Papo

***Local Clinical Research unit:*** Estelle Marcault, Marhaba Chaudhry, Charlène Da Silveira, Annabelle Metois, Ismahan Mahenni, Meriam Meziani, Cyndie Nilusmas

***Local Clinical Research team:*** Sylvie Le Gac, Awa Ndiaye, Françoise Louni, Malikhone Chansombat, Zelie Julia, Solaya Chalal, Lynda Chalal

***Pharmacy:*** Laura Kramer, Jeniffer Le Grand

***Biological Resource Center***: Kafif Ouifiya, Valentine Piquard, Sarah Tubiana

**French COVID cohort study group**

***Principal investigator:*** YazdanYazdanpanah, Hôpital Bichat Claude Bernard, AP-HP, Paris, IAME INSERM UMR1137, Université Paris Diderot

***Scientific Manager:*** Xavier Duval Centre d’investigation clinique 1425, Hôpital Bichat Claude Bernard, AP-HP, Paris IAME INSERM UMR1137, Université Paris Diderot

***Methodology :*** France Mentré, Cédric Laouénan, Jade Ghosn, Centre d’investigation clinique 1425, Hôpital Bichat Claude Bernard, AP-HP, ParisIAME INSERM UMR1137, Université Paris Diderot

The biobank French-Covid was stored in CRB Paris Saclay (BRIF: BB-0033-00089).
