# Supplementary material for: More rapid blood interferon α2 decline in fatal versus surviving COVID-19 patients
Source: Front Immunol. 2023 Nov 21;14:1250214. doi: 10.3389/fimmu.2023.1250214 (PMC10703045; doi:10.3389/fimmu.2023.1250214)
Supplement: Supplementary file 2 [file Image_1.pdf]

Manuscript Title: More rapid blood interferon α2 decline in fatal versus surviving COVID-19 patients. Supplementary Materials:

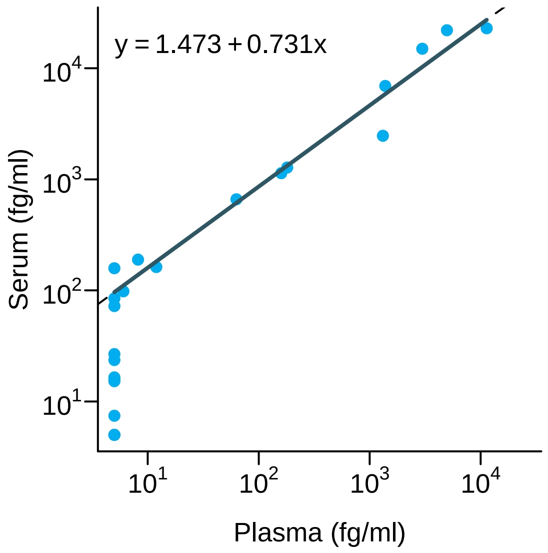

Fig. S1: **Correlation between the dosage of IFN-α2 in serum and plasma**. The equation allows to convert (log_{10}) plasma concentrations to (log_{10}) serum concentrations, for values above 5 fg/ml. On the scale of serum concentrations, the limit of quantification was therefore 5 fg/mL for IFN-α2 with dosage in the serum, but 96.37 fg/mL for IFN-α2 with dosage in the plasma.

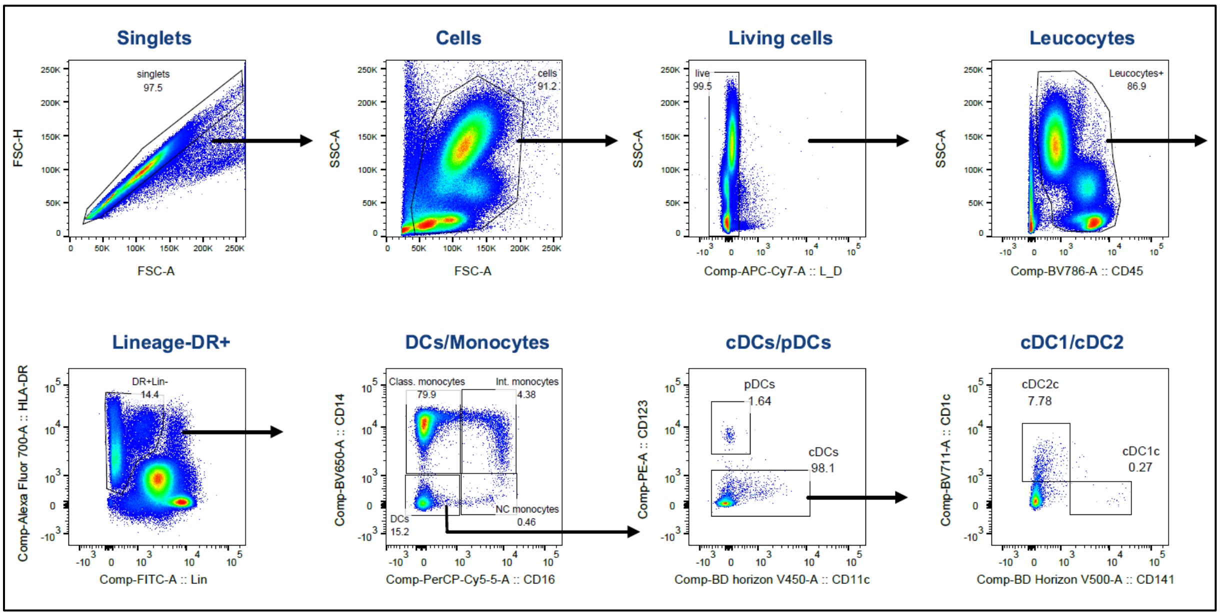

Figure S2: Flow cytometry gating strategies for evaluating pDC, cDC1 and cDC2.

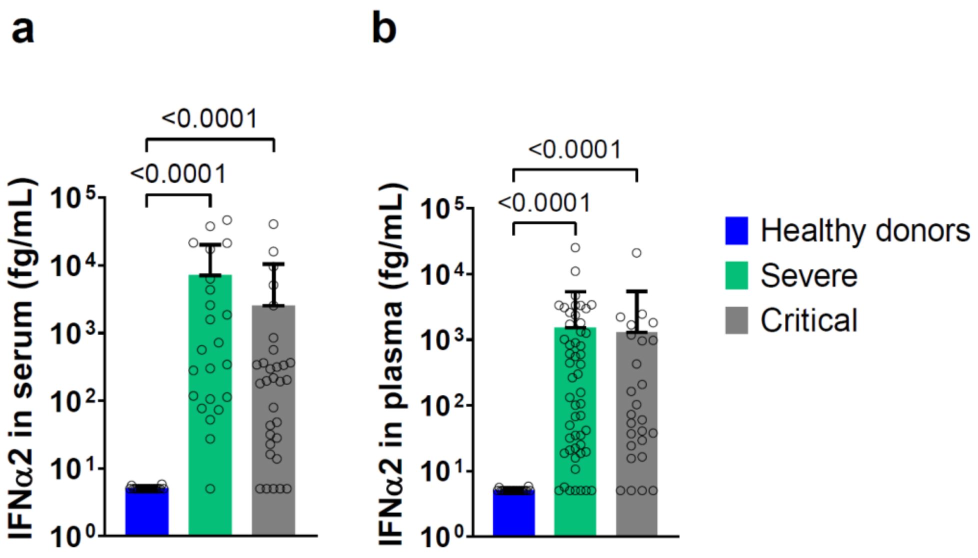

Fig.S3: Mean blood IFN-α2 level at D1 in severe and critical COVID-19 patients, and in healthy donors in plasma and serum (**1a**) Detection of IFN-α2 in serum only. (**1b**) Detection of IFN-α2 dosage in serum only. (**1c)** Detection of IFN-α2 dosage in plasma only.

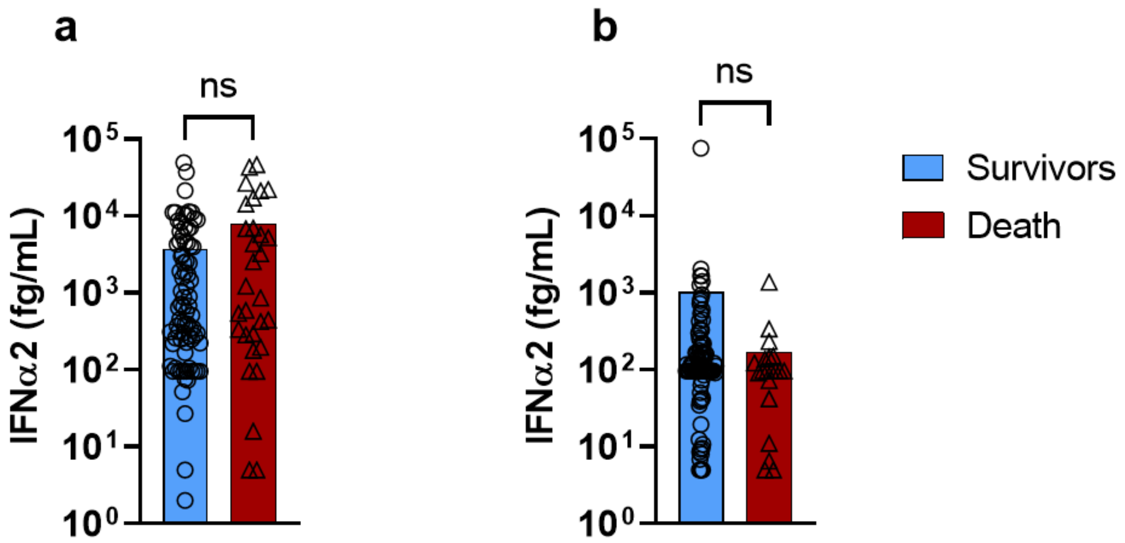

Fig. S4: Blood IFN-α2 levels at D1 and D6 in survivors and patients who will subsequently die. Bars represent the mean IFN-α2 levels in fg/mL in survivors (blue, n=104) and dead patients (dark red, n=28) at D1 **(S2a)** and at D6 **(S2b)** (*Mann-Whitney's test*).

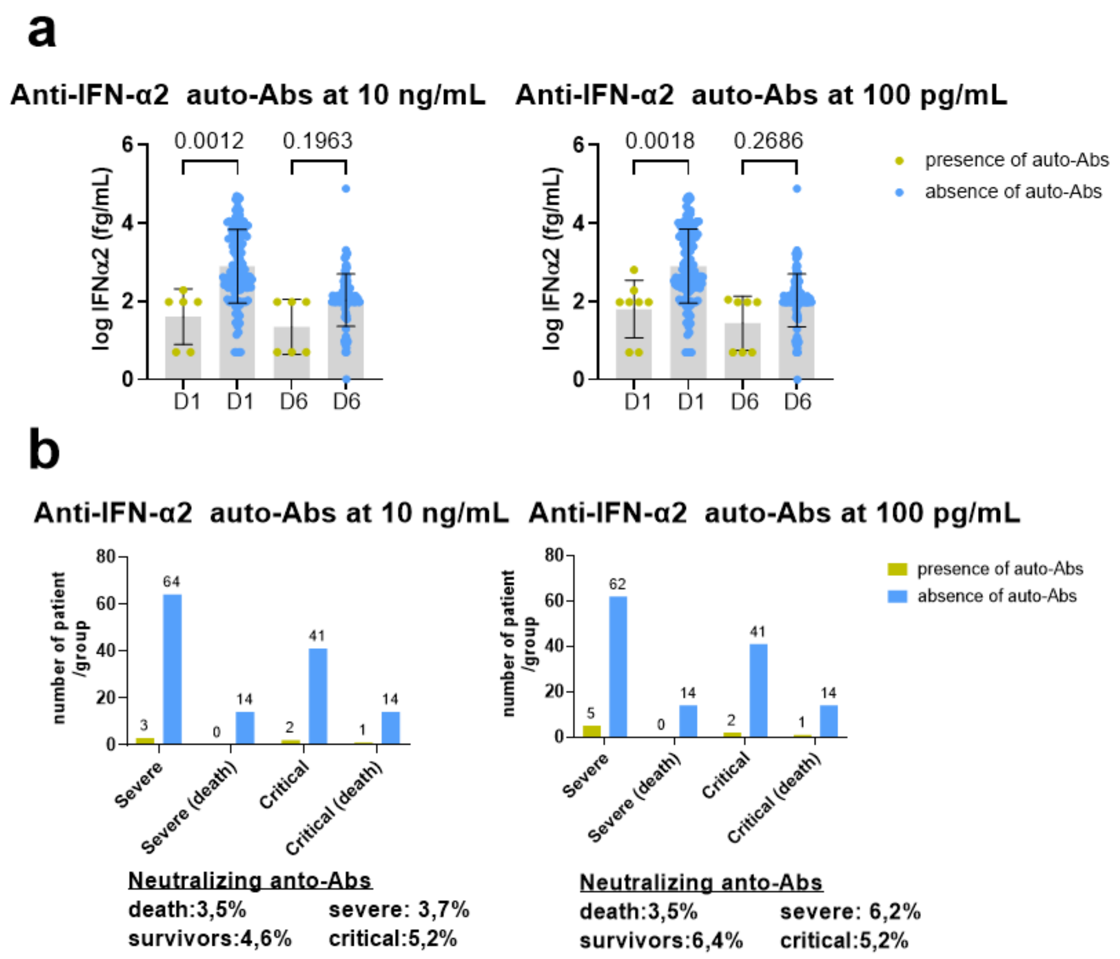

Fig. S5: Anti-IFN-α2 auto-Abs measurements in patients with severe and critical COVID-19 and correlation with the circulating levels of IFN-α2 dosage. (S5a) Results of IFN-α2 dosage at D1 and D6 in blood at 10 ng/mL (left) and 100 pg/mL (right). Bars represent the mean level of IFN-α2 in fg/mL for each group: auto-Ab positive patients (green dots or bar) and auto-Ab negative patients (blue dots or bar), and symbols represent individual measures (*One-way ANOVA, followed by Tukey's post-test*). **(S5b)** Number of anti-IFN-α2 auto-Ab positive patients at 10 ng/mL (left) and 100 pg/mL (right) in the severe or critical patients, compared between patients who survived or died.

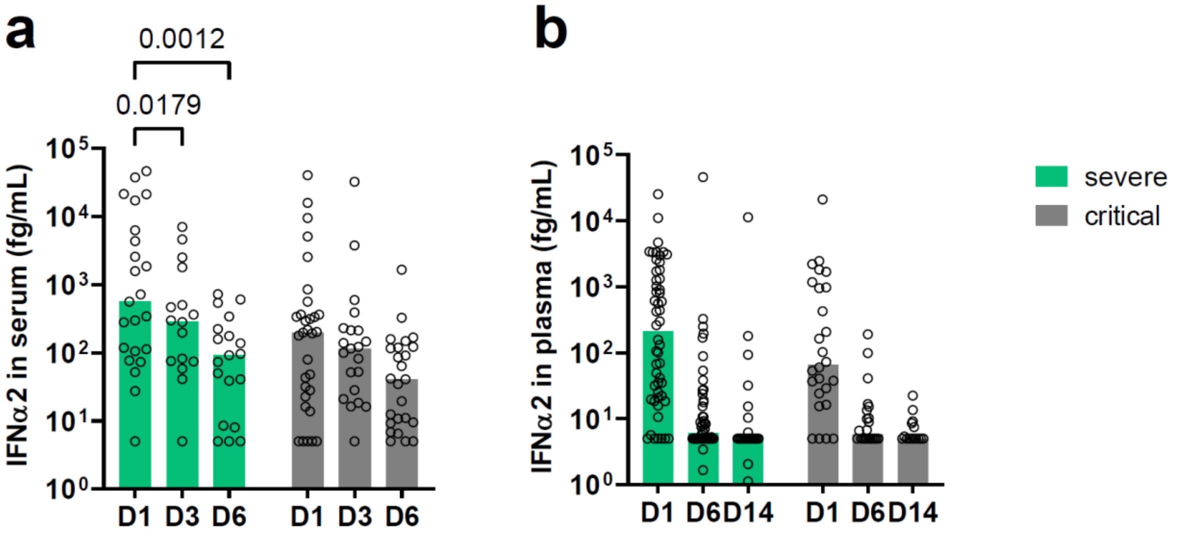

Fig. S6: Results of serum and plasma IFN-α2 dosage in severe and critical patients over time. Bars represent the mean level of IFN-α2 in fg/mL for each group: severe patients (green bar) and critical patients (light grey bar), and symbols represent individual measures. **(S5a)** Results of IFN-α2 dosage at D1, D3 and D6 in serum. Serum IFN-α2 waned between D1 and D3 and D6 in severe patients (*Two-way ANOVA, followed by Sidak's post-test*). **(S5b)** Results of IFN-α2 dosage at D1, D6 and D14 in plasma (*Two-way ANOVA, followed by Sidak's post-test*).

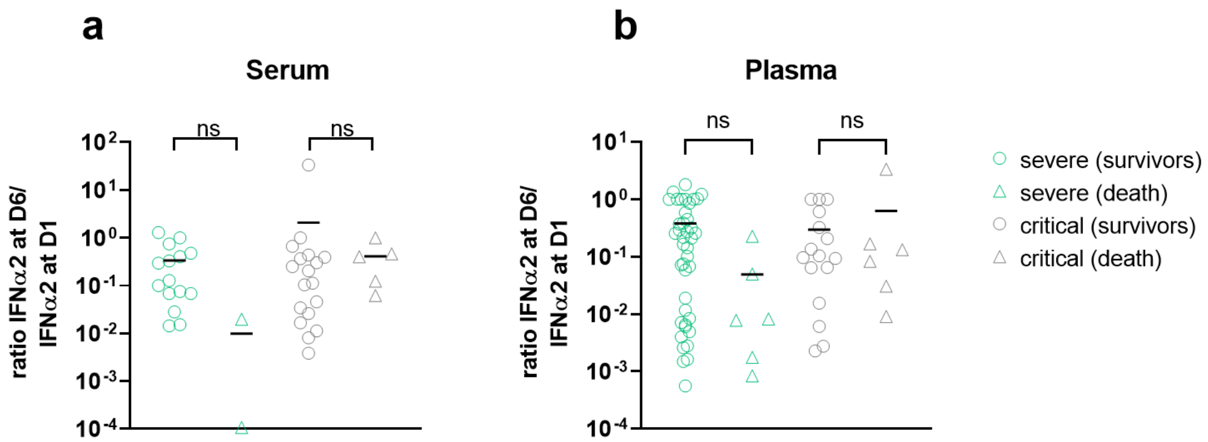

Fig. S7: D6/D1 ratio of serum and plasma IFN-α2 level in severe patients and critical patients. Symbols represent individual measures: severe patients (green symbols, n=17 for serum, n= 47 for plasma) and critical patients (light grey symbols, n=23 for serum, n=22 for plasma). The lines represent the mean ratios; patients who subsequently died are represented with triangles and survived patients in circles. **(S6a)** Ratios of serum FN-α2 level in severe patients and critical patients. **(S6b)** Ratios of plasma FN-α2 level in severe patients and critical patients. Among the severe patients, those who perished depicted lower ratios IFN-α2 D6/ IFN-α2 D1 than those who survived (for serum and plasma data sets) (*Kruskal-Wallis's test followed by Dunn's post-test*).
